# Moral distress and moral injury among nurses in German acute inpatient care – a cross-sectional study

**DOI:** 10.1186/s12912-026-05526-7

**Published:** 2026-10-17

**Authors:** Tobias Mai, Anna-Henrikje Seidlein, Michael Schilder, Iulia Dahmer

**Affiliations:** 1 Nursing Research, Nursing Department, University Hospital, Goethe University Frankfurt, Frankfurt am Main, Germany; 2 Institute of Health Sciences, Chair for Nursing Science, University Medicine Rostock, Schillingallee 70, 18057 Rostock, Germany; 3 Evangelische Hochschule Hessen, Protestant University of Applied Sciences, Darmstadt, Germany; 4 Institute of Biostatistics and Mathematical Modelling, Faculty of Medicine, Goethe University Frankfurt, Frankfurt am Main, Germany

**Keywords:** Nursing, Moral suffering, Moral distress, Moral injury, Organizational support, Organizational culture, Moral integrity

## Abstract

**Background:**

Moral suffering in health care is increasingly a focus of research. The extent of and relationship between two distinct forms of moral suffering, namely, moral injury (MI) and moral distress (MD), has not yet been studied in nurses in acute inpatient care in Germany.

**Objectives:**

This study aims to examine the severity of MI and MD in the context of organizational support, the correlation between the two constructs, and the variables associated with the extent of MD and MI.

**Methods:**

We conducted a cross-sectional study including 1323 nurses employed in German hospitals. The data were collected in March and April 2023 in an online survey using the German version of the Moral Distress Scale (MDS), the German Moral Injury Symptom Scale for Health Professionals (G-MISS-HP), the Perceived Organizational Support Scale (POS-s) and an additional questionnaire on the individual perception of ethical and structural conditions in the institution. The data were analyzed descriptively. Partial credit Rasch models were fitted to the item-level responses of the G-MISS-HP and the MDS to obtain interval-level measures of moral injury and moral distress for subsequent analyses. Median regressions with backward variable selection were performed for MD- and MI-Rasch scores to identify variables associated with the level of moral distress/injury. The associations between the MD and MI-Rasch scores were tested using Spearman’s correlation test.

**Results:**

A total of 68.6% (*n* = 907; 95% CI: 66.1%–71.1%) of the respondents scored above the validated G-MISS-HP screening threshold (≥ 29), which has previously been associated with impairment in family, social, and occupational functioning. Spearman’s correlation analysis revealed statistically significant positive associations between the MD-Rasch score and the MI-Rasch subscores (guilt/shame subscore: *r* = 0.57, 95% CI [0.53; 0.61]; spiritual troubles subscore: *r* = 0.22, 95% CI [0.16; 0.27]; condemnation subscore: *r* = 0.39, 95% CI [0.34; 0.43], all *p* < 0.001). Median regression analyses revealed significant associations of both MD and MI with perceived organizational support (POS-s), satisfaction with the treatment team’s consideration of own perspective, responsibility for staff, and participation in interprofessional case discussions.

**Conclusions:**

Manifestations of moral suffering are common. Our findings highlight the importance of measures to promote an ethical climate and to address the negative consequences of moral suffering, particularly at the management level.

**Registration:**

German Clinical Trials Register (https://www.drks.de/): DRKS00030785 (date: 06 February 2023).

**Supplementary Information:**

The online version contains supplementary material available at https://doi.org/10.1186/s12912-026-05526-7.

## What is already known

• Nurses experience moral suffering because of the complex ethical situations they face.

• Moral suffering can lead to health problems or the ability to leave the profession.

**What this paper adds**

• Symptoms of clinically relevant moral injury were frequently reported by acute care nurses.

• Higher levels of moral distress were strongly associated with higher levels of moral injury symptoms.

• Organizational support of and consideration for nurses’ professional perspectives were associated with lower levels of moral distress and moral injury symptoms, highlighting these factors as potential targets for future interventions.

**Supplementary Information:**

The online version contains supplementary material available at https://doi.org/10.1186/s12912-026-05526-7.

## Background

Research on moral distress (MD) has expanded to an extensive body of empirical knowledge, especially regarding nurses, as the milestone definition by Andrew Jameton states that “Moral distress arises when one knows the right thing to do, but institutional constraints make it nearly impossible to pursue the right course of action” [1]. A correlation between MD and the intention to leave the profession has been reported for nurses [2–4]. The initial definition has also been developed conceptually several times. Moreover, numerous narrower and broader definitions of MD exist [5, 6].

Particularly in the context of the coronavirus pandemic, another phenomenon, moral injury (MI), which has, to date, played a rather subordinate role in the context of health care, has also increasingly come into focus [7–9]. The conceptual origin of MI can be traced back to the military and its approach to medicine; it has only recently found its way into the field of health care. The particular importance of MI for health-related outcomes, such as posttraumatic stress disorder, depression, and substance abuse, has already been demonstrated, especially in military samples [10, 11]. MI entails a profound psychological burden resulting from actions that violate personal moral or professional ethical principles. This also includes refraining from actions that would be appropriate and necessary according to one’s own moral standards [12, 13]. Although it was originally described in military settings, the concept is increasingly considered relevant to nursing. Nurses in acute inpatient care are often confronted with ethically challenging situations, including resource constraints, perceived inadequate care, conflicts between professional values and organizational demands, witnessing care that they consider morally wrong, and being placed in situations where they are unable to act in accordance with their professional and ethical responsibilities. These characteristics make nurses particularly vulnerable to persistent moral conflict and may help explain why moral distress can evolve into moral injury among some individuals.

The distinction between MD and other related concepts, particularly MI, poses a challenge to nursing practice and research [14, 15]. Both MD and MI focus on different types of moral suffering, which are difficult to objectify because they are very personal experiences that are perceived differently by individual. They violate individuals’ conscience and moral integrity and evoke strong moral feelings. Nevertheless, there is conceptual ambiguity regarding the qualitative content and conceptual boundaries of the theories and explanatory models. This is not only a problem in terms of research methodology. It is also important for clinical assessments and for developing preventive, ethical and (psycho-)therapeutic interventions.

Although Grimell and Nilsson (2020) attempted to integrate the two concepts for military psychology [16], whether and to what extent they can be applied to nursing professionals is currently unclear. These two constructs have also recently been theorized as stages of moral escalation [17]. The specific relationship between MI and MD among nurses in acute inpatient care has not yet been the subject of empirical studies in Germany. Such empirical and theoretical groundwork has direct consequences for clinical ethics work that is intended to support nurses in dealing with and overcoming moral challenges. Many methods (e.g., educational programs and consultation services) have been (further) developed and tested to prevent and reduce MD [18–20]. These include approaches such as “reflective debriefing” [21]. The extent to which specific communication structures may also prevent MI is unclear.

This study assessed the severity of moral distress and moral injury, examined the association between both constructs, and identified individual and organizational factors associated with their levels among nurses in acute inpatient care in Germany. Perceived organizational support refers to employees’ general belief that their organization values their contributions and cares about their well-being [22]. This concept is rooted in Organizational Support Theory, which suggests that employees develop global perceptions concerning the extent to which the organization supports them. Greater perceived organizational support is associated with stronger organizational commitment, job satisfaction, and employee engagement.

## Methods

The present study represents the quantitative component of an explanatory sequential two-stage mixed-methods project. It was followed by a qualitative substudy that aimed to clarify and empirically differentiate the unexplained aspects of MD and MI.

The study investigated the following research questions:

1. How severe are MD and MI among nurses in German acute-care hospitals?
2. Is there a correlation between the extent of MD and MI?
3. a. Which factors are associated with the severity of MI?
  b. Which factors are associated with the severity of MD?

### Setting and procedure

Data were collected using an online self-report questionnaire via a cross-sectional SoSciSurvey. Recruitment was conducted through professional nursing societies, professional associations, and institutional networks, including the German University Hospital Network VPU (Verband der Pflegedirektorinnen und Direktoren der Universitätskliniken). As invitations were disseminated through multiple distribution channels, the total number of eligible recipients was unknown; consequently, a response rate could not be calculated. Participants had to fulfill the following inclusion criteria: registered nurse, working in acute care at a hospital in Germany, sufficient knowledge of the German language to answer the questions (based on participants’ self-assessment), and the ability to provide informed electronic consent. The data collection took place in March and April 2023.

The German versions of the Moral Distress Scale (MDS), the German Moral Injury Symptom Scale for Health Professionals (G-MISS-HP), the Perceived Organizational Support Scale (POS-S), and an additional questionnaire on the individual perception of ethical and structural conditions in the institution were included in the survey. The order of the instruments used to assess MD and MI was randomized so that any influence of the respective preceding questionnaire could be tested.

A pretest with 15 nurses working in different fields of acute inpatient care was conducted to evaluate the clarity and comprehensibility of the questionnaire items and to identify potential problems in wording or interpretation.

### Sample size

A sample of at least 1238 completed surveys was used. This sample size was considered sufficient for identifying an effect of 0.015 (Cohen’s f²) with 80% power at a significance level of 5% when a multiple linear regression with a maximum of 14 covariates was performed. Multiple linear regressions for interval-scaled measures of MI and MD (estimated using Rasch models) were planned, but median (quantile) regressions were performed instead because the data violated the linear model assumptions. Given the large size of the sample, the power was still considered adequate for the more robust model (median regression).

The questionnaire was made up of 24 pages. Page 24 contained only an optional to collect an email address for follow-up contact and was not required for study participation. Only questionnaires that reached at least Page 23, thereby completing all mandatory study questions, were exported and included in the analyses. The restriction to questionnaires reaching Page 23 was defined a priori during data export to ensure that all mandatory study variables were available for analysis.

### Outcome measures

For the severity of MD, MI and perceived organizational support, the following scales were used:

- The German Moral Injury Symptom Scale for Health Professionals (G‑MISS-HP) incorporates 9 items, with total scores ranging from 9 to 90. The items were assessed using a 10-point Likert scale ranging from 1 (complete disagreement) to 10 (complete agreement), with 5 indicating neutrality. The response scale included repeated verbal anchors, as defined in the original G-MISS-HP instrument. Its internal consistency is good (Cronbach’s α = 0.79; Guttman’s λ2 = 0.80). Factor analysis supported a three-factor structure, and the scale demonstrates convergent validity with the German version of the Second Victim Experience and Support Tool (G‑SVESTR). Scores above the optimal cutoff of 28.5 points indicate a clinically relevant level of moral injury, indicating potential impairment in family, social, and occupational functioning [23]. Decisions based on this cutoff score provide a sensitivity of 89% and a specificity of 63% [24].
- The German version of the Moral Distress Scale (MDS) includes 12 items, with separate subscale scores for intensity and frequency each ranging from 0 to 48. The intensity of MD was assessed using a 5-point Likert scale ranging from 0 (none) to 4 (very high). Frequency was measured using a 5-point Likert scale ranging from 0 (never) to 4 (more than once a week). Previous psychometric evaluations using Rasch analysis indicated that the items are reliable and that the scale is well targeted, although the Cronbach’s α for the final German version was not explicitly reported [25]. The scale was validated for measuring MD among nurses in acute care settings.
- The Perceived Organizational Support–Short Scale (POS-S) consists of 8 items, with total scores ranging from 8 to 56. Responses were measured using a 7-point Likert scale ranging from 1 (strongly disagree) to 7 (strongly agree), with 4 indicating neutrality. The internal consistency indices were very good (Cronbach’s α = 0.91; McDonald’s ω = 0.91). Convergent validity is supported by a significant positive correlation with general self-efficacy (*r* = 0.15, *p* < 0.05), whereas criterion validity is indicated by a significant negative correlation with resistance to change (*r* = − 0.18, *p* < 0.01) [22].
- Contextual variables related to demographics and working conditions were also recorded using a self-developed questionnaire (Supplement 1).

### Ethical reflection

Nurses have specialized knowledge because of their professional training, which includes learning how to address moral challenges. However, even if nurses per se do not represent a vulnerable population, the actual, individual, and situation-specific vulnerability of individual participants may deviate from this general assessment [26]. Individual nurses, for example, could be particularly vulnerable because of previous personal experiences or life circumstances or because of a lack of professional support in coping with morally distressing events. Accordingly, the research team considered numerous strategies for preventing and, if necessary, dealing with stressful events that could have been triggered by study participation. If necessary, one of the authors, a trained ethics consultant, was able to offer a supportive discussion. The study was approved by the Institutional Review Board at University Medicine Greifswald (BB 168/22a). The study is registered in the DRKS (DRKS00030785 (date: 06 February 2023)) (https://www.drks.de/).

### Statistics

The statistical analysis was performed with IBM SPSS Statistics, version 31.0 and R 4.5.1 (R Foundation for Statistical Computing, Vienna, Austria), the *TAM* (version 4.3–25), *pROC* (version 1.19.0.1) and *quantreg* (version 6.1) packages. The data were analyzed using descriptive statistics: for categorical variables, absolute and relative frequencies were reported, and continuous variables were described using medians, interquartile ranges, means and standard deviations.

Respondents who reported symptoms of moral injury, defined as clinically relevant impairment in daily life resulting from moral suffering, were classified as having MI symptoms if they scored above the published G-MISS-HP cutoff of 28.5 points [24]. Because only integer raw scores are possible, a cutoff of ≥ 29 was applied, corresponding to the published threshold of 28.5. The proportion of respondents with MI symptoms was reported as a percentage with a 95% confidence interval (CI).

The effect of the order in which MDS and G-MISS-HP were presented in the questionnaire on the presence/absence of MI symptoms was tested via the chi-square test.

Analysis of the severity of MI and MD based on the G-MISS-HP scores and the MDS scores (using the scores of the intensity items, which indicate the level of disturbance), respectively, was performed using Rasch models. Because the questionnaires consisted of items with polytomous response categories, partial credit Rasch models were used. These are probabilistic models belonging to the class of latent trait item response theory models. Unlike classical test theory, which treats all items as equally informative, Rasch models allow items to have different difficulties that are then considered when the total or partial score of the questionnaire is constructed. Moreover, these models account for the fact that the probability of a particular response depends on both the respondent’s ability (level of moral distress/injury in our case) and the item difficulty. This results in interval-level person estimates that more accurately reflect the underlying construct and provide a more robust outcome measure for subsequent analyses (such as identifying influencing variables and estimating associations) than using raw questionnaire scores alone does.

Fitting the Rasch models to the data allows us to estimate the levels of moral distress/injury of the respondents as one or several interval-scaled latent traits that incorporate the item difficulties. On the basis of [25], where one-dimensionality of the German version of the MDS was reported, we fitted a one-dimensional Rasch model to the data from this questionnaire and estimated one latent trait quantifying the level of moral distress (MD-Rasch score). Using the three-factor structure of the G‑MISS-HP ([24]), a three-dimensional Rasch model was fitted to the G‑MISS-HP data, and three latent traits quantifying the guilt/shame factor (Items 1–4), the spiritual troubles factor (Items 5–7) and the condemnation factor (Items 8 and 9) (resulting in three MI-Rasch subscores) were estimated.

The MD- and MI-Rasch scores were used for all further analyses.

Univariate logistic regression was performed to examine the association between the MD-Rasch score and the proportion of respondents who reported symptoms of MI (binary: above vs. below 29 points in the raw score). Spearman’s correlation was used to assess the association between the MD and MI-Rasch scores. On the basis of the escalation model [16, 17], an ROC analysis was conducted to evaluate the performance of the MD-Rasch score as a marker for identifying subjects with MI symptoms. An optimal cutoff for the MD-Rasch score was selected according to the Youden index, and the corresponding raw MDS score was reported.

Median regressions with backward variable selection based on the Akaike information criterion (AIC) were performed for the MD-Rasch score and the three MI-Rasch scores to identify variables associated with the severity of MD and MI.

Drawing on previous research, the following variables were considered potential covariates: participation in ethics consultations (1 = yes), availability of short-term support (1 = yes), participation in interprofessional case discussions (1 = yes), participation in nursing rounds (1 = yes), satisfaction with one’s own perspective during planned team communication (1 = yes), availability of ethical guidelines (1 = yes), staff absences during the last shift (1 = no), professional experience (years), part-time employment (1 = yes), responsibility for staff (1 = yes), perceived organizational support (mean POS-S score), field of practice (general medical/surgical ward [reference category], intensive/intermediate care, psychiatric nursing, emergency department, perioperative nursing [e.g., operating room, anesthesia], and other), responsibility for childcare (< 18 years) (1 = yes), and responsibility for caring for dependent persons in private life (1 = yes). The selection of these variables was informed by previous studies on moral distress, organizational support, and double-duty caregiving [27–35]. Koenker and Machado’s pseudo-R^2,^ which measures the relative success of median regression in terms of an appropriately weighted sum of absolute residuals, was reported as a goodness-of-fit measure for the median regression [36]. No adjustment for multiple testing was applied because the regression analyses were considered exploratory and hypothesis-generating.

Despite the large sample size, linear regression models were not an optimal choice for the analyses, as the residual distribution was skewed to the right and presented outliers, and the assumption of normally distributed residuals was not met. Normality was assessed using the Shapiro–Wilk test. A significance level of 5% was used for all analyses.

## Results

The questionnaire URL was accessed more than 6000 times during the study period. Given that this number reflects webpage accesses rather than the full contribution of individual participants, it would include repeated visits and technical accesses. After the eligibility criteria (e.g., employment in acute inpatient care) were applied and the dataset was restricted to questionnaires that completed all mandatory study pages (last page ≥ 23), the final analytical sample included 1323 complete datasets. Table 1 provides an overview of the sample.

### Characteristics of MI and MD

A total of 68.6% (CI [66.1%; 71.1%]) of the respondents (*n* = 907) in the sample had a G-MISS-HP score ≥ 29 points and, thus, a relevant expression of MI for limitations in everyday life. In the overall sample, the median G-MISS-HP score was 35 (Q1 = 26–Q3 = 45; range: 9–78). The order of the questionnaires (G-MISS-HP or MDS first) showed no statistically significant correlation with the severity of MI ≥ 29 points (x² = 0.408; *p* = 0.554). The intensity and frequency of MD indicate a high level of exposure to morally distressing situations. Three items showed particularly high levels of intensity of MD (Table 2): shift staffing (55%), operational and financial pressure (39%), and insufficient competence among colleagues (intra- and interprofessional) (39%). Half of the respondents experienced these situations once or more per week. The median MDS score of the intensity items was 30 (Q1 = 23, Q3 = 36; range: 1–48).

**Table 1:** Descriptive statistics of the sample characteristics

	*N*=	1323
	*n*	%
*Gender*		
*Male*	352	26.6
*Female*	949	71.7
*Other*	8	0.6
*No answer*	14	1.1
*Age*		
< 29 years	246	18.6
30–39 years	356	26.9
40–49 years	313	23.7
50–59 years	319	24.1
> 60 years	89	6.7
*Private life situation (multiple choice)*		
Living alone	278	21.0
Living with child/children	399	30.2
Living with partner	913	69.0
Living in shared apartment	55	4.2
Responsible for childcare (< 18 years)	375	28.3
Responsible for people in need of nursing care	231	17.5
*Highest qualification*		
3-year course of nursing	561	42.4
Further training in nursing	508	38.4
Academic degree (Bachelor’s, Master’s, Diploma, Doctorate)	244	18.4
No answer	10	0.8
*Professional experience*		
< 4 years	161	12.2
5–9 years	224	16.9
10–19 years	319	24.1
20–29 years	305	23.1
> 30 years	314	23.7
*Employment*		
Full-time employment	814	61.5
Part-time employment	487	36.8
no answer	22	1.7
*Responsibility for staff*		
Yes	328	24.8
No	976	73.8
No answer	19	1.4
*Field of practice*		
General medical/surgical ward	395	29.9
Intensive/intermediate care	497	37.6
Psychiatric nursing	98	7.4
Emergency department	81	6.1
Perioperative nursing (e.g., operating room, anesthesia)	127	9.6
other	125	9.4
*Patients*		
Adult care	1202	90.9
Pediatric care	121	9.1
*Ward size*		
Up to 10 beds	148	11.2
Up to 20 beds	401	30.3
Up to 30 beds	385	29.1
Up to 40 beds	139	10.5
More than 40 beds	65	4.9
Not relevant because of special field	185	14.0
*Hospital size*		
Up to 100 beds	67	5.1
Up to 300 beds	230	17.4
Up to 500 beds	197	14.9
Up to 700 beds	130	9.8
Up to 1000 beds	167	12.6
More than 1000 beds	465	35.1
o not know	67	5.1

**Table 2:** Intensity and frequency of moral distress assessed with the MDS (Moral Distress Scale) (*n* = 1323)

	mean	SD	median	intensity					mean	SD	median	frequency				
				0	1	2	3	4				0	1	2	3	4
				none	low	average	high	very high				never	less than once a month	monthly	weekly	several times a week
Was unable to deliver care in keeping with my professional ethical principles due to adminstrative or budgetary pressure.	3.0	1.0	3	33	81	252	444	513	2.9	1.2	3	43	178	204	393	505
				(2%)	(6%)	(19%)	(34%)	(39%)				(3%)	(13%)	(15%)	(30%)	(38%)
Have experienced patients or family members being given “false hope”, contrary to what is known about their situation.	2.6	1.2	3	107	150	296	380	390	2.0	1.1	2	109	372	390	295	157
				(8%)	(11%)	(22%)	(29%)	(29%)				(8%)	(28%)	(29%)	(22%)	(12%)
Have carried out physician’s orders for tests or treatments that I considered to be inappropriate in view of the treatment goal.	2.5	1.3	3	117	169	313	370	354	2.2	1.2	2	104	314	354	367	184
				(9%)	(13%)	(24%)	(28%)	(27%)				(8%)	(24%)	(27%)	(28%)	(14%)
Have taken no action when a member of the multidisciplinary team made a medical error and failed to report it.	1.6	1.6	1	532	140	213	200	238	0.7	0.8	1	631	518	129	32	13
				(40%)	(11%)	(16%)	(15%)	(18%)				(48%)	(39%)	(10%)	(2%)	(1%)
Have had to provide care to patients for which I felt unqualified, resulting in quality of care being diminshed or patients being put at risk.	1.7	1.6	1	478	191	173	202	279	0.9	1.0	1	516	526	173	69	39
				(36%)	(14%)	(13%)	(15%)	(21%)				(39%)	(40%)	(13%)	(5%)	(3%)
Have worked with nurses or other health-care providers I felt were lacking in competence, resulting in the quality of care being diminished or patients being put at risk.	2.9	1.1	3	56	101	278	374	514	2.5	1.2	3	54	273	316	349	331
				(4%)	(8%)	(21%)	(28%)	(39%)				(4%)	(21%)	(24%)	(26%)	(25%)
Have taken no action in instances where there were signs of possible verbal or physical abuse* of patients or patient neglect**.	1.7	1.6	2	514	138	163	247	261	0.9	1.0	1	596	463	152	85	27
				(39%)	(10%)	(12%)	(19%)	(20%)				(45%)	(35%)	(11%)	(6%)	(2%)
Have seen the quality of care and treatment suffer due to a lack of provider continuity within the treatment team.	2.7	1.1	3	71	143	313	446	350	2.5	1.2	3	59	238	320	376	330
				(5%)	(11%)	(24%)	(34%)	(26%)				(4%)	(18%)	(24%)	(28%)	(25%)
Have worked on a nursing team with staffing levels that I considered inadequate.	3.2	1.1	4	63	72	151	304	733	2.8	1.2	3	63	163	267	308	522
				(5%)	(5%)	(11%)	(23%)	(55%)				(5%)	(12%)	(20%)	(23%)	(39%)
Because of uncollegial collaboration I was unable to deliver a level of care in keeping with my professional ethical principles.	2.4	1.3	3	178	182	277	369	317	1.8	1.2	2	176	414	330	253	150
				(13%)	(14%)	(21%)	(28%)	(24%)				(13%)	(31%)	(25%)	(19%)	(11%)
I was unable to prevent or alleviate the suffering of a patient due to inadequate physician’s orders.	2.6	1.4	3	168	126	207	385	437	1.7	1.1	2	171	479	342	228	103
				(13%)	(10%)	(16%)	(29%)	(33%)				(13%)	(36%)	(26%)	(17%)	(8%)
I have had to informally assume responsibility for work done by other hospital workers.	2.1	1.4	2	242	196	286	332	267	2.0	1.4	2	231	288	257	299	248
				(18%)	(15%)	(22%)	(25%)	(20%)				(17%)	(22%)	(19%)	(23%)	(19%)

*Patients were deliberately physically, emotionally or mentally abused, frightened or put at risk

**Patient’s needs were deliberately not met or required services were not performed even though the necessary resources were available

SD = standard deviation

### Associations between MI-Rasch scores and MD-Rasch scores

The estimated Rasch scores showed a monotonically increasing correspondence to the raw scores: higher Rasch scores indicated higher raw scores and therefore higher levels of MD and MI. The Rasch analysis indicated that the respondents could differentiate between the response options, resulting in ordered thresholds for both the MD and the three MI subscores. The person separation index (PSI), which measures the ability of an instrument to distinguish between respondents of different trait levels, was very high for MD (0.88) and ranged from very high to poor for the three dimensions of the MI model (0.81, 0.65, 0.43). Similarly, model reliability was also high for the MD-Rasch model (0.83) and ranged from good to poor for the MI-Rasch model (0.70, 0.35, 0.44). Local independence was largely supported, with only one item pair in the MD-Rasch model exceeding the criterion of 0.30 above the average residual correlation and no such pairs in the MI model. The residual-based item-fit statistics (unweighted (outfit) and weighted (infit) fit mean square) were within acceptable limits (Supplement 2).

Spearman’s correlation revealed statistically significant positive associations between the MD-Rasch score and the MI-Rasch subscores (guilt/shame subscore: *r* = 0.57, 95% CI [0.53; 0.61]; spiritual troubles subscore: *r* = 0.22, 95% CI [0.16; 0.27]; condemnation subscore: *r* = 0.39, 95% CI [0.34; 0.43], all *p* < 0.001).

**Fig. 1:**
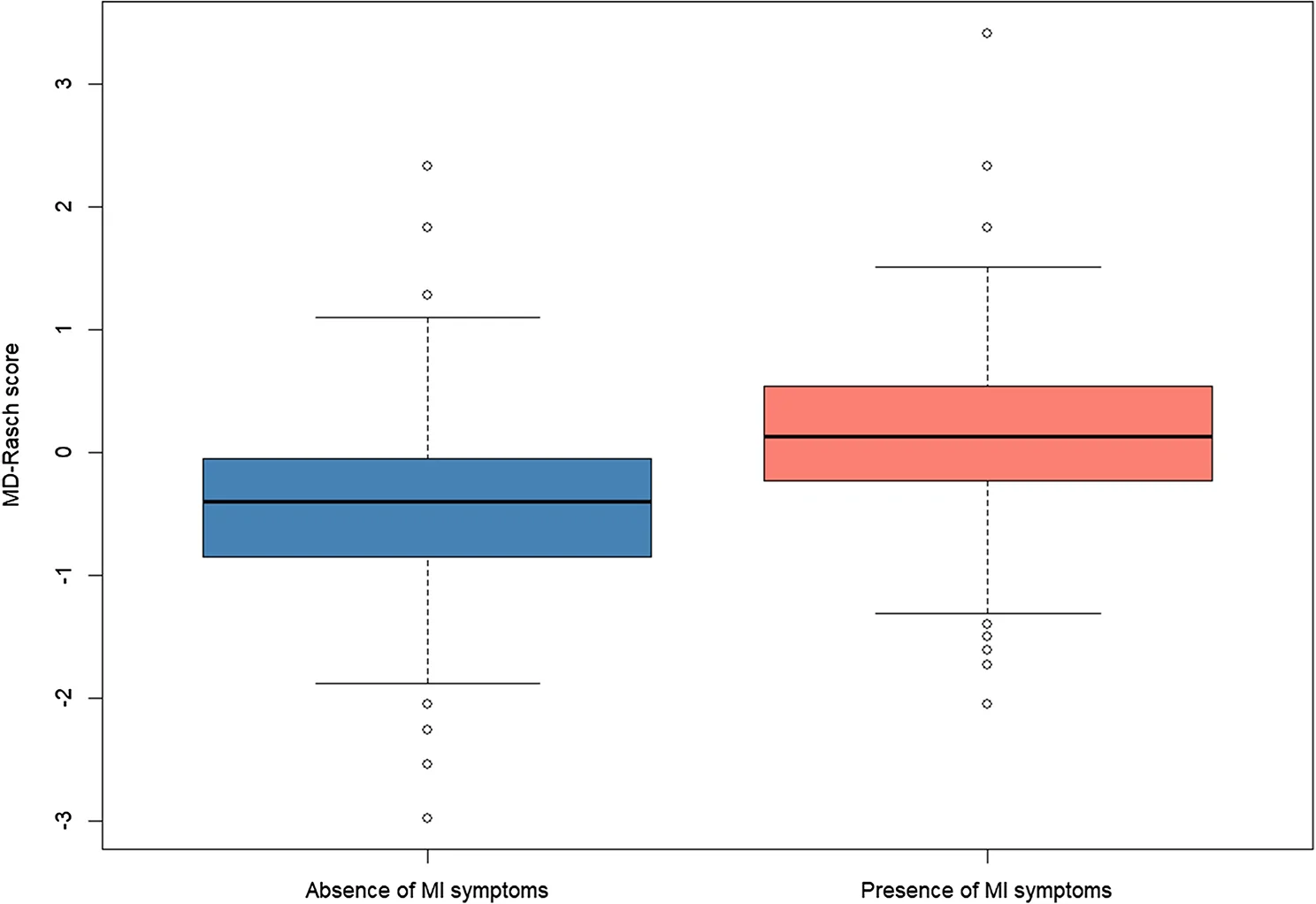
MD-Rasch scores across respondents exhibiting MI symptoms (G-MISS-HP scores below/above 29 points)

Univariate logistic regression indicated that higher MD-Rasch scores were significantly associated with higher odds of moral injury symptoms according to the G-MISS-HP classification (*p* < 0.001). Specifically, a one-unit increase in the MD-Rasch score was associated with 5.26-fold greater odds of moral injury symptoms (OR = 5.26, 95% CI [4.17; 6.72]) (Fig. 1). The MD-Rasch score ranged from − 2.98 to 3.41, a one-unit increase in the score corresponding to approximately one-seventh of the scale. The fit of the model was significantly better than that of the null model (*p* < 0.001), and the Hosmer–Lemeshow test revealed no evidence of inconsistency between the observed and fitted values (*p* = 0.263).

**Fig. 2:**
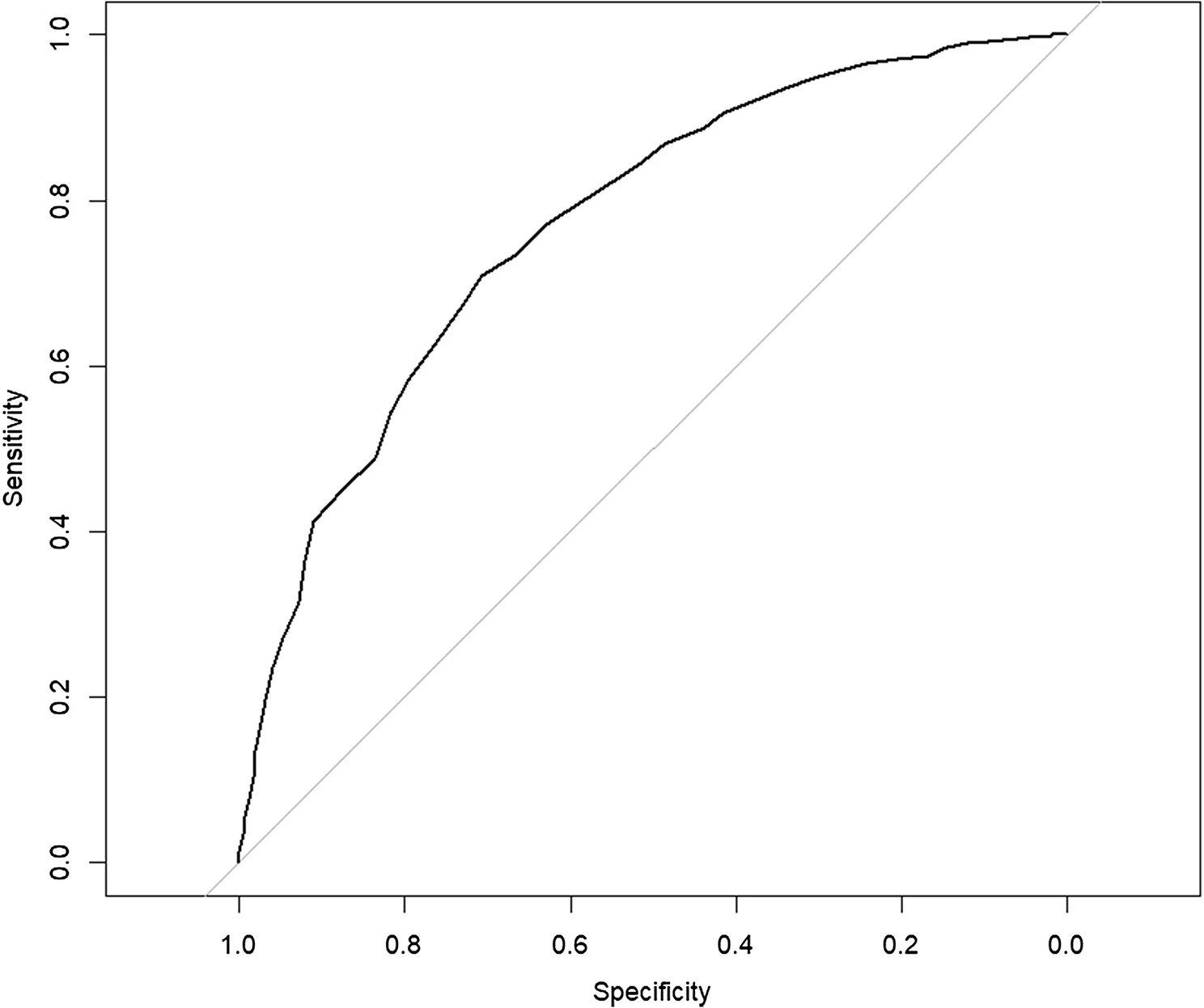
ROC curve for evaluating the performance of the MD-Rasch score as a marker for identifying subjects with MI symptoms

ROC analysis (AUC = 0.771, 95% CI [0.744; 0.798]) revealed an optimal raw MDS cutoff of ≥ 27 points for discriminating between participants classified below versus at or above the G-MISS-HP screening threshold (sensitivity 71%, 95% CI [64%; 79%], specificity 71%, 95% CI [62%; 77%]) (Fig. 2).

### Variables associated with the MI-Rasch score and MD-Rasch score

Reported access to organizational support services included interprofessional case discussions (54.8%), peer consultation (54.5%), ethics case discussions (44.5%), supervision (26.5%), nursing rounds (21.5%), de-escalation specialists (15.2%), and ethics guidelines (11.9%).

The variables retained after backward selection in the median regression analyses are presented in Table 3. The mean POS-S score was 3.37 (SD = 1.358, 95% CI 3.30–3.45), which was below the scale midpoint, indicating relatively low perceived organizational support. Lower perceived organizational support was significantly associated with higher MD-Rasch scores and higher MI-Rasch subscores. Greater satisfaction with the extent to which the treatment team considered one’s perspective was significantly associated with lower MD and MI-Rasch subscores. In contrast, experiencing short-term staff absence was significantly associated with higher MD-Rasch scores and higher MI-Rasch subscores for Factors 1 (guilt/shame) and 2 (spiritual troubles). Having responsibility for staff significantly correlated with lower MI-Rasch subscores for Factors 1 and 2. Working in perioperative nursing was associated with lower MD-Rasch scores than working on general medical or surgical wards were. Finally, lower MI-Rasch subscores for Factor 3 (condemnation) were associated with greater work experience and a lack of responsibility for a dependent person in private life.

**Table 3:** Results of median regression analyses of MD- and MI-Rasch scores

Dependent variable	Variables*	*(n* = *1323)*	
		Regression coefficient, 95% CI	*p*
MI - Rasch Factor 1	Participation in interprofessional case discussions (1 = yes)	0.072 (0.01, 0.135)	**0.023**
(guilt/shame)	Satisfied with considerations one’s own point of view in planned team communication (1 = yes)	-0.176 (-0.241, -0.112)	**< 0.001**
	Staff absences in the last shift (1 = no)	-0.139 (-0.271, -0.006)	**0.04**
	Professional experience in employment (in years)	-0.002 (-0.004, 0.001)	0.157
	Responsibility for staff (1 = yes)	-0.098 (-0.179, -0.016)	**0.019**
	Perceived organizational support (mean POS-s)	-0.105 (-0.128, -0.081)	**< 0.001**
MI - Rasch Factor 2	Participation in ethics consultations (1 = yes)	-0.07 (-0.121, -0.019)	**0.007**
(spiritual troubles)	Short term support available (1 = yes)	-0.032 (-0.077, 0.013)	0.168
	Participating nursing rounds (1 = yes)	-0.031 (-0.082, 0.02)	0.236
	Satisfied with considerations one’s own point of view in planned team communication (1 = yes)	-0.099 (-0.141, -0.057)	**< 0.001**
	Staff absences in the last shift (1 = no)	-0.079 (-0.153, -0.005)	**0.037**
	Part-time employment (1 = yes)	-0.03 (-0.069, 0.009)	0.133
	Responsibility for staff (1 = yes)	-0.059 (-0.104, -0.015)	**0.01**
	Perceived organizational support (mean POS-s)	-0.035 (-0.051, -0.02)	**< 0.001**
	Field of practice: intensive/intermediate care	-0.013 (-0.059, 0.034)	0.595
	Field of practice: psychiatric nursing	0.071 (-0.023, 0.166)	0.141
	Field of practice: emergency department	-0.062 (-0.143, 0.019)	0.135
	Field of practice: perioperative nursing (e.g., operating room, anesthesia)	-0.005 (-0.07, 0.061)	0.886
	Field of practice: other	0.038 (-0.029, 0.106)	0.266
MI - Rasch Factor 3	Satisfied with considerations one’s own point of view in planned team communication (1 = yes)	-0.159 (-0.211, -0.107)	**< 0.001**
(condemnation)	Staff absences in the last shift (1 = no)	-0.038 (-0.085, 0.01)	0.119
	Professional experience in employment (in years)	-0.005 (-0.007, -0.003)	**< 0.001**
	Responsibility for staff (1 = yes)	-0.043 (-0.095, 0.009)	0.108
	Responsibility for caring for dependent persons in private life (1 = yes)	0.08 (0, 0.159)	**0.049**
	Perceived organizational support (mean POS-s)	-0.063 (-0.081, -0.045)	**< 0.001**
MD - Rasch	Participation in ethics consultations (1 = yes)	0.1 (-0.013, 0.213)	0.082
	Short term support available (1 = yes)	-0.093 (-0.193, 0.008)	0.071
	Participating nursing rounds (1 = yes)	-0.106 (-0.22, 0.008)	0.069
	Satisfied with considerations one’s own point of view in planned team communication (1 = yes)	-0.172 (-0.26, -0.084)	**< 0.001**
	Staff absences in the last shift (1 = no)	-0.23 (-0.372, -0.088)	**0.002**
	Professional experience in employment (in years)	-0.003 (-0.007, 0.0004)	0.066
	Perceived organizational support (mean POS-s)	-0.159 (-0.19, -0.128)	**< 0.001**
	Field of practice: intensive/intermediate care	0.043 (-0.057, 0.143)	0.401
	Field of practice: psychiatric nursing	0.017 (-0.178, 0.212)	0.864
	Field of practice: emergency department	-0.019 (-0.148, 0.11)	0.774
	Field of practice: perioperative nursing (e.g., operating room, anesthesia)	-0.267 (-0.435, -0.099)	**0.002**
	Field of practice: other	0.023 (-0.149, 0.195)	0.792

MI = Moral Injury; MD = Moral Distress

* Variables kept after the backward selection based on AIC

All four regression models were significantly better fits than the respective null-models (all *p* < 0.001). The values of the pseudo-R1 measures for the median regressions with MD-Rasch score and the MI-Rasch subscores were 0.11, 0.07, 0.07 and, respectively 0.08. Multicollinearity of the predictor variables was assessed (all correlations below 0.35)

## Discussion

The present study revealed that 68.6% of participants screened positive for clinically relevant moral injury symptoms according to the validated G-MISS-HP screening threshold. The median MDS score was 30 (Q1 – Q3: 23–36; range: 1–48), reflecting substantial moral distress in this self-selected sample. Greater moral distress was positively associated with moral injury symptoms, although the strength of the associations varied across the three MI dimensions. Furthermore, organizational factors, including perceived organizational support and consideration of nurses’ professional perspectives during team communication, were associated with both moral distress and moral injury symptoms. The field of practice was not associated with either outcome, except for perioperative nursing. One possible explanation for this is that shorter and less continuous patient contact in perioperative settings may reduce exposure to ethically challenging situations. A similar mechanism may also help explain the association between responsibility for staff and lower MI levels for Factors 1 and 2, as leadership roles may involve greater decision-making autonomy and opportunities to address moral conflicts. However, these interpretations should be considered exploratory.

The mean G-MISS-HP score in our study was higher than that reported by Trifunovic-König et al. [24], whose multiprofessional sample included only 33% nurses. These findings support previous findings suggesting that nurses may be particularly affected by moral injury symptoms, as has also been shown for MD [37].

While some studies report lower MD levels, they consistently emphasize the need for adequate coping strategies and supportive work environments [38–40]. In this study, higher MD levels were associated with higher odds of screening positive for clinically relevant moral injury symptoms. These findings are consistent with those of previous studies reporting a close relationship between moral distress and moral injury [16, 41, 42].

ROC analysis revealed that the exploratory MDS cutoff of ≥ 27 points achieved a sensitivity of 71% and a specificity of 71% compared with the G-MISS-HP screening classification. Thus, approximately 29% of participants who were classified above the G-MISS-HP screening threshold were not identified by the selected MDS cutoff. These findings support the conceptual distinction between moral distress and moral injury and highlighted their close empirical association. One possible explanation is that the perception of distress in morally challenging situations may diminish over time, whereas moral injury symptoms may persist. Alternatively, some morally injurious experiences may not be adequately captured by the construct of moral distress [14]. The observed association between moral distress and moral injury could support the hypothesis of an escalation pathway; however, this interpretation remains theoretical and hypothesis-generating. Given the cross-sectional design, our findings cannot establish whether moral distress precedes or develops into moral injury. Integrating the present quantitative results with insights from a qualitative interview study [43] may help clarify these aspects.

The observed associations between organizational factors and both moral distress and symptoms of moral injury are consistent with previous research highlighting the importance of organizational support, communication, and ethical work environments [30–32, 39, 44–46]. Perceived organizational support and consideration of one’s own perspective in team communication were significantly associated with both MD and MI levels. The promotion of interprofessional perspective-taking may help reduce moral distress among health care professionals [29]. In contrast, our findings did not show an association between participation in interprofessional case discussions and moral distress. Instead, participation was associated with higher MI subscale 1 scores and participation in interprofessional ethics consultations with higher MI subscale 2 scores. One possible explanation is that discussing ethically challenging situations may increase awareness of moral transgressions and feelings of guilt rather than immediately alleviating moral injury-related symptoms. Furthermore, staff absence during the last shift was significantly associated with higher MD and higher MI for subscale 1 and 2 scores (Guilt/Shame, Spiritual Troubles). More than 89% of the respondents reported staff shortages during their last shift, highlighting the practical relevance of these findings. Situations involving insufficient or inadequately qualified staff occurred at least weekly for more than half of the participants. MD related to staffing shortages was higher than in comparable samples from Switzerland and German outpatient care [25, 47], although comparability is limited because of contextual differences. Moreover, the prevalence of sickness-related absences among care professionals in Germany has steadily increased [48]. These developments may contribute to the elevated moral suffering observed and are alarming. Staffing-related issues were also identified as major stressors in a Swedish study [49], which reported higher MD scores among nurses with additional qualifications. This may also apply to the present sample, which included a high proportion of intensive care nurses and academically trained staff. Greater professional qualifications may increase moral sensitivity, making ethical conflicts more salient [50], whereas years of professional experience were associated with lower scores on the condemnation subscale of moral injury. While the present study revealed no association with moral distress but only with moral injury, Alshammari et al. reported a similar association for moral distress [34]. This discrepancy may be explained by differences in the study setting. In their study, which was conducted among emergency department nurses, unexpected and acute clinical situations are central to daily practice and may therefore have a stronger influence on moral distress.

In the present study, several potential targets for intervention were identified. Our findings emphasize the importance of including individual perspectives in team communication as a means of preventing or coping with moral suffering. However, supportive communication structures such as nursing rounds or ethics case discussions were underutilized. Thus, in addition to structural measures, individual interventions to strengthen moral resilience should be implemented [42]. Regular reflection formats, such as case discussions or ethics consultations, may support coping with ethical challenges and reduce MD [51, 52]. However, only approximately half of the respondents reported access to such formats.

The effectiveness of ethics consultations depends on the willingness of participants, particularly leaders, to engage in these processes [53]. The strong association between perceived organizational support and both MD and MI levels indicates a need for action at the organizational level. Creating time and space for moral reflection (“moral space”) and facilitating structured discussions may strengthen ethical competence and reduce moral distress over time [42, 54]. Leadership plays a key role in establishing these organizational conditions. Taken together, our findings and the recent evidence discussed above suggest that leadership behavior represents an important organizational resource for fostering an ethical climate by enhancing perceived organizational support and ethical reflection, thereby creating conditions that may contribute to reducing moral distress and the risk of moral injury among nurses.

## Limitations and strengths

The use of a convenience sample limits generalizability. Selection bias is possible, as more burdened nurses may have been more likely to participate. In addition, reliance on self-reported data introduces the risk of common method bias. A further limitation is the lack of complete participation-flow data for the online survey, including the number of questionnaire starts, early withdrawals, and intermediate participation stages, which limits a detailed assessment of potential attrition and selection bias.

The original sample size calculation was performed for multiple linear regression, but because the outcome distributions violated the model assumptions, more robust models (median regressions) were chosen for the analyses, which represents a minor limitation. Nevertheless, due to its large sample, the study retains substantial statistical precision, although the power differs slightly between the two regression models. Because no adjustment for multiple testing was applied, the reported associations should be interpreted with caution and require confirmation in future studies. Furthermore, the pseudo-R² values ranged from 0.07 to 0.11, indicating that the regression models explained only a modest proportion of the outcome variation. Accordingly, the identified organizational factors should be interpreted as contributing factors, not comprehensive explanations of moral distress or moral injury, as a substantial proportion of the variation remains unexplained.

The distinction between MD, general work-related stress, and MI may be blurred, potentially leading to overestimation [55]. However, this is not a problem that is specific to our study; rather, it stems from the lack of clarity regarding the constructs and, consequently, their theoretical basis in the instruments. In addition, the relatively low reliability and person separation values observed for MI Factors 2 and 3 indicate limited differentiation between respondents. Findings involving these subscales may therefore be less stable and should be interpreted with caution. The planned integration of quantitative and qualitative findings may help to address this issue.

Owing to the cross-sectional design, no conclusions can be drawn regarding temporal or causal relationships between moral distress and moral injury. Furthermore, although the results contribute to clarifying the relationship between MD and MI, they do not allow conclusions regarding their prevalence among hospital nurses in general.

A major strength of the study is its large sample size (*n* = 1323). Randomizing the order of the MDS and G-MISS-HP instruments minimized potential response bias. Owing to potential interruptions while nurses completed the survey (e.g., patient care demands), participants may have restarted the questionnaire later. As no unique identifier, device restriction, or IP-based control was used, repeated participation by the same individual could not be completely excluded.

## Implication for practice

Nurses often must navigate between patient needs, interprofessional perspectives, organizational demands, and personal moral values. Subsequent balancing or negotiation could lead to moral burden. Owing to the extent of MD and MI, detecting and handling such experiences is important. Organizations, especially nursing leaders, must offer safe spaces for debriefing such constraining situations.

## Conclusion

Nursing in complex patient situations and the challenge of scarce resources in health care may lead to increasing moral conflicts. Consequently, supporting health care professionals in addressing these experiences remains an important challenge. Organizations need to recognize this responsibility by providing structures that enable open communication, ethical reflection, and discussion of morally challenging experiences. A shift from blaming low personal resilience toward shared organizational responsibility is needed to prevent moral injury as a potential consequence of unresolved morally distressing events. Interventions to enhance moral resilience should be viewed as one component of a broader organizational strategy that includes supportive leadership, ethical reflection, and structural improvements rather than as a substitute for organizational responsibility.

## Supplementary Information

Below is the link to the electronic supplementary material.

Supplementary Material 1

## Data Availability

The datasets generated and analyzed during the current study are not publicly available because participants’ consent was based on the condition that the data would not be shared beyond the study team.

## Declarations

### Declarations

This study was conducted in accordance with the Declaration of Helsinki.

## Abbreviations

AIC: Akaike information criterion
AUC: Area under the curve
CI: Confidence interval
G-MISS-HP: German Moral Injury Symptoms Scale for Health care Professionals
MD: Moral Distress
MDS: Moral Distress Scale
MI: Moral Injury
OR: Odds ratio
POS: Perceived Organizational Support
ROC: Receiver Operating Characteristics
PSI: Person separation index
URL: Uniform Resource Locator
