# Supplementary material for: Moral distress and moral injury among nurses in German acute inpatient care – a cross-sectional study
Source: BMC Nurs. 2026 Oct 17;25:990. doi: 10.1186/s12912-026-05526-7 (PMC13676794; doi:10.1186/s12912-026-05526-7)
Supplement: Supplementary file 1 [file 12912_2026_5526_MOESM1_ESM.docx]

**Supplements**

**Supplement 1: additional questionnaire**

The questionnaire assessing demographic characteristics and work-related contextual factors was originally developed and administered in German. The English translation provided in the supplement was prepared by the authors for publication purposes.

1. **Number of beds in the hospital where you are currently employed:**

*(Free-text response (number of beds))*

Recoding for statistical analyses:

ordinal categorical variable: *hospital size*

1. up to 100 beds	=1

1. up to 300 beds	=2

1. up to 500 beds	=3

1. up to 700 beds	=4

1. up to 1000 beds	=5

1. more than 1000 beds	=6

1. don't know	=7

2. **Current area of practice:**

- General medical/surgical ward
- Intensive care unit (including intermediate care)
- Psychiatric nursing
- Emergency department
- Perioperative nursing (e.g., operating room, anesthesia)
- Other (please specify)

3. **Size of the ward/unit where you are employed:**

- Up to 10 beds
- Up to 20 beds
- Up to 30 beds
- Up to 40 beds
- More than 40 beds
- Not applicable (functional unit)

4. **On my ward/unit, I predominantly care for:**

- Children/adolescents
- Adults

5. **In my ward/unit, clinical ethics consultation is available to me as a registered nurse (i.e., ethical case consultations provided by qualified ethics consultants).**

- Yes

Recoding for statistical analyses:

Binary variable: *Participate in ethics case consultations*

5. No	= 0

5. I do not know	= 0

6. No	= 0

6. Yes	= 1

- No
- I do not know

6. **I participate in these ethics case consultations.**

- Yes
- No

7. **Which options for short-term or low-threshold support are available to you during or after stressful events on your ward/unit?**
   *(Multiple responses were permitted. Participants selecting at least one support option were coded as 1 ("support available"). Participants selecting "None" were coded as 0.)*

- De-escalation trainers or experts

Recoding for statistical analyses:

Binary variable: *availability of short-term support*

7. None	= 0

7. At least one support option selected	= 1

- One-to-one patient observation staff (observation pool)
- Peer consultation
- Chaplaincy or spiritual care
- Supervision
- None
- Other (please specify) (Free-text response)

8. **Interprofessional case discussions (e.g., registered nurses, physicians) are conducted on my ward/unit.**

- Yes, regularly
- Yes, irregularly (as needed or upon request)

Recoding for statistical analyses:

Binary variable: *participation in interprofessional case discussions*

8. No	= 0

8. I do not know	= 0

9. No	= 0

6. Yes	= 1

- No
- I do not know

9. **I participate in these interprofessional case discussions**

**(e.g., registered nurses, physicians).**

- Yes
- No

Recoding for statistical analyses:

Binary variable: *participation in nursing rounds*

10. No	= 0

10. I do not know	= 0

10. Yes, I can participate	= 1

10. Yes, I can initiate them	= 0

10. **Nursing rounds take place on my ward/unit.**
    *(Multiple responses possible)*

- Yes, I can participate
- Yes, I can initiate them
- No
- I do not know

11. **How satisfied are you with the extent to which your professional perspective is considered in planned communication within the treatment team (e.g., during daily rounds)?**

- Very satisfied

Recoding for statistical analyses:

Binary variable: *satisfaction with the consideration of one’s own point of view in planned team communication*

11. very satifsfied	= 1

11. somewhat satisfied	= 1

11. neither satisfied nor dissatisfied	= 0

11. somewhat dissatisfied	= 0

11. very dissatisfied	= 0

- Somewhat satisfied
- Neither satisfied nor dissatisfied
- Somewhat dissatisfied
- Very dissatisfied

12. **On my ward/unit, ethical guidelines provide guidance for professional practice (e.g., decision-making regarding the use of restraints, consideration of a child’s wishes).**

- Yes

Recoding for statistical analyses:

Binary variable: *availability of ethical guidelines*

12. yes	= 1

12. no	= 0

12. I do not know	= 0

- No
- I do not know

13. **If yes, which ethical guidelines are available on your ward/unit?**
    (Please specify) *(Free-text response)*
14. **During my most recent shift on my ward/unit, staff shortages occurred.**

Binary variable: *staff absences during the last shift*

14. yes	= 1

14. no	= 0

- Yes
- No

**15. During my most recent shift on my ward/unit, overcapacity occurred**

(i.e., more patients required care than specified by the ward’s bed capacity and staffing plan)

- Yes
- No

16. **Sociodemographic Characteristics**

**Please provide some information about yourself.**

Age (in years) *(Free-text response)*

Gender:

- Male
- Female
- Diverse
- Prefer not to say

17. **What is your highest vocational or academic qualification in nursing?**

- 3-year nursing training program
- further training in nursing
- academic degree (Bachelor’s, Master’s, Diploma, Doctorate)
- Prefer not to say

18. **How many years have you been working in nursing since completion of your professional qualification?**
    (Periods such as parental leave or caregiving leave are not included.)
    Years of professional nursing experience *(Free-text response)*
19. **Workload in direct patient care:**
    My current weekly workload is:

Recoding for statistical analyses:

Binary variable: *part-time employment*

19. full-time	= 0

19. Up to 75% full-time equivalent	= 1

19. Up to 50% full-time equivalent	= 1

- Full-time
- Up to 75% full-time equivalent
- Up to 50% full-time equivalent
- Prefer not to say

20. **Do you hold one or more additional professional qualifications (e.g., specialist nursing certification)?**

- No
- Yes
- Prefer not to say

21. **I hold the following additional professional qualification(s):**
    (Multiple responses and free-text answers possible)

- Palliative care
- Pain management / Pain nurse
- Stroke nurse
- Lactation and breastfeeding counseling
- Oncology nursing
- Anesthesia and/or intensive care nursing
- Psychiatric nursing
- Ethics consultation
- Practice supervision / clinical education
- Wound care specialist
- Other (please specify) (Free-text-response)

22. **Do you hold a leadership position with personnel responsibility?**

Binary variable: *responsibility for staff*

22. yes	= 1

22. no	= 0

- Yes
- No
- Prefer not to say

23. **Living situation:**
    (Multiple responses possible)

- Living alone
- Living with child/children
- Living with partner
- Living in a shared apartment
- Prefer not to say

24. **In addition to my nursing profession, I am responsible for:**
    (Multiple responses possible)

- Responsibility of care for child/ children < 18 years
- Responsibility for people in need of nursing care
- Not applicable
- Prefer not to say

Recoding for statistical analyses:

Binary variable: *responsibility for childcare*

24. Responsibility of care for child/ children < 18 years	= 1

Recoding for statistical analyses:

Binary variable: *responsibility for caring for dependent persons in private life*

24. Responsibility for people in need of nursing care	= 1

**Supplement 2 : Goodness-to-fit measures for the Rasch models**

|  | MDS Rasch model | MISS Rasch model | | |
| --- | --- | --- | --- | --- |
|  |  | Guilt/shame subscore | Spiritual troubles subscore | Condemnation subscore |
| **Measure** | | | | |
| Item fit (range) |  | | | |
| Infit | 0.89 - 1.27 | 0.83 – 1.33 | 0.98 – 0.99 | 0.96 – 1.09 |
| Outfit | 0.86 - 1.34 | 0.83 – 1.36 | 0.97 – 0.98 | 0.93 – 1.05 |
| Reliability | 0.83 | 0.70 | 0.35 | 0.44 |
| Separation index | 0.88 | 0.81 | 0.65 | 0.43 |
| Local independence  Items with residual correlation more than 0.3 above average | 1 pair of items  (MDS2, MDS3, 0.34 above average) | none | | |
| Ordered thresholds | yes | yes | | |
